# Self Assembly of Nano Metric Metallic Particles for Realization of Photonic and Electronic Nano Transistors

**DOI:** 10.3390/ijms11052242

**Published:** 2010-05-25

**Authors:** Asaf Shahmoon, Ofer Limon, Olga Girshevitz, Zeev Zalevsky

**Affiliations:** 1 School of Engineering, Bar-Ilan University, Ramat-Gan 52900, Israel; E-Mail: asaf.sh11@gmail.com (A.S.); 2 Nanotechnology Center, Bar-Ilan University, Ramat-Gan 52900, Israel

**Keywords:** nanotechnology, van der Waals forces, intra molecular self-assembly

## Abstract

In this paper, we present the self assembly procedure as well as experimental results of a novel method for constructing well defined arrangements of self assembly metallic nano particles into sophisticated nano structures. The self assembly concept is based on focused ion beam (FIB) technology, where metallic nano particles are self assembled due to implantation of positive gallium ions into the insulating material (e.g., silica as in silicon on insulator wafers) that acts as intermediary layer between the substrate and the negatively charge metallic nanoparticles.

## 1. Introduction

Nano metric particles with different shapes and sizes have been attracting major attention in the past few years, due to their both unique optical as well as electronic characterization [1]. The field of nano particles can be divided into two basic parallel research directions: the research field that deals with analyzing the physical and chemical properties of the nano particles as a function of their size, shape, internal morphological, dielectric constant and aspect ratio [2–4], and the research field that deals with the self assembly of the nano metric particles.

Self assembly of nano metric particles into patterned structures has a large variety of multidisciplinary functionality and applicability in fields such as engineering [5–7], photonics [8], chemistry [9] and material sciences, in realizing various types of nano metric passive as well as active devices such as nano metric electronic or photonic transistors, nano photonic hyper spectral sensors, analyzers of concentration of chemical compositions and nano particles for enhancing the imaging resolution of microscopes [10].

Two basic approaches for constructing nano and micro scale devices are “top down” and “bottom up” techniques. The method we present in this paper, for the construction of well defined arrangements of self assembly nano metric particles, combines these two approaches. At first, we create the desired pattern using the focused ion beam (top down approach) and, finally, we deposit the nano metric particles on a surface of our substrate in order to create the self assembly organization into the desired patterns (bottom up approach).

Several methods have been employed to produce self assembly of nano metric particles on a surface using: DNA as a framework for arrangement of metallic nano components [11,12], evaporation induced self assembly (EISA) techniques [13] or a method whereby carbon-coated transmission electron microscopy (TEM) copper grid is acting as substrate material [14]. Other methods for deposition of nano metric particles at predetermined areas on a surface of a substrate were performed using an atomic force microscope (AFM) tip in order to apply pulses of voltage into insulating films [15,16], by printing technique that used electrostatic force to pattern PMMA (polymethyl methacrylate) [17], by electrostatic force generation of *p-n* junctions [18] or by lithography process that used conductive flexible poly-dimethyl-siloxane (PDMS) stamps to charge the substrate [19].

In this paper, we present a new approach for constructing a well defined arrangement of self assembly nano metric gold (Au) particles in sophisticated nano structures. The concept is based upon Focused Ion Beam (FIB) technology [20]. In Section 2, we describe the self assembly method for constructing the well defined arrangement of the nano metric particles. In Section 3, we present preliminary experimental results for possible types of devices. The paper is concluded in Section 4.

Please note that in contrast to research mentioned in [5], which focuses on applications of having nano particles at specific areas on the chip, in this paper we focus on the self assembly property of the proposed method as well as the chemical considerations that are required in order to enable the self assembly of the nano metric particles. In addition, in contrast to [5], here we also pay attention to various new devices that can benefit from the self assembly property (e.g., the electronic nanotransistor).

## 2. Self Assembly Method

The self assembly of nano metric particles into well defined area is performed by accelerating concentrated positive gallium ions (Ga^+^) into an insulating material. Note that as described by [20], during the FIB operations, a small amount of positive gallium ions are implanted in the sample, and large numbers of secondary electrons leave the sample. Those gallium ions etch off any exposed surface. Gallium ions will also be implanted into the top few nanometers of the surface, and therefore will create the seeds later on to attract the nano particles according to their predetermined pattern, while controlling the self assembly process. Implantation of positive gallium ions on the surface generates positively charge patterns that act as an intermediary layer between the surface and the nano metric particles. The theoretical self assembly resolution is determined by the FIB sputter limit for the signal to noise ratio which is of about 6 nm.

**Figure 1.**
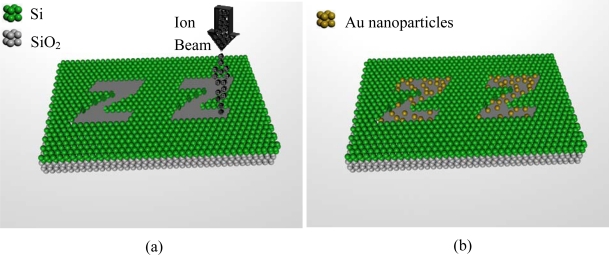
Atomic sketch of the system. **(a)** Generation of a charged pattern using focused ion beam (top down approach). **(b)** Deposition of Au nanoparticles in order to obtain the self assembly process (bottom up approach).

In Figure 1, we present an atomic sketch of the system, which demonstrates the top down (Figure 1(a)) and bottom up (Figure 1(b)) approaches that are required to obtain the self assembly.

After installing the nanoparticles into the predefined nano metric structures, we obtain tunability of those devices by applying external electronic or photonic control command that rearranges the position of those nanoparticles inside the devices, and by this affects (*i.e.*, modulates) the propagating stream of an information signal (that can as well be either electronic or photonic). The core of this self assembly technique can be extended to other methods, such as e-beam lithography, in order to create negatively charged patterns that will later on create the basis for attracting positively charged nano particles. Nonetheless, the main advantage of the FIB technique is related, among others, to its ability to achieve a very high precision self assembly, which meanwhile could not be achieved by other methods (e.g., e-beam lithography).

## 3. Experimental Results and Devices

In the experiments, we used unconjugated gold colloidal in an aqueous suspension (SPI supplies). The overall net charge on the gold colloidal is negative. The diameters of the particles that we used were either 200 nm or 30 nm, while the standard deviation of the size distribution was less than 10%. The nano particles were kept at 4 °C, and before the self assembly process we brought them to room temperature. Please note that the basis for the technology that permits the manufacture of stable suspensions (e.g., one in which the particles do not clump), without a stabilization coating, is the overall net charge on the colloidal particle surfaces is negative, and this provides the mechanism by which particles repel one another and the suspension remains stable.

**Figure 2.**
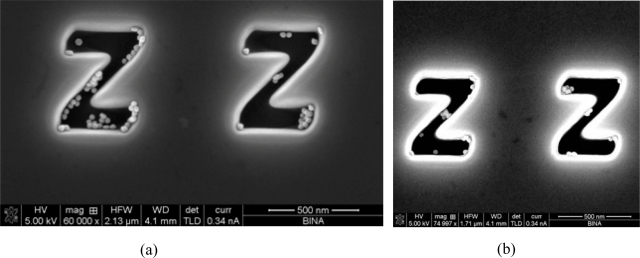
SEM images of the fabricated “ZZ” pattern after the self assembly process. **(a)** The “ZZ” pattern with tunnel width of 150 nm. **(b)** The tunnel width is 90 nm.

In Figure 2, we present scanning electron microscope (SEM) images of the self assembly process of the nano metric particles at a unique “ZZ” pattern. The tunnel width of the “ZZ” pattern in Figure 2(a) is about 150 nm, while in Figure 2(b) the tunnel width is 90 nm. The pattern was created using FIB having the following parameters: ion beam voltage of 30 kV and a beam current of 1.5 pA. One may see how indeed the nano metric particles self assemble according to the pattern generated by the FIB. Note that the ability to control the positively charge patterns, where the self assembly is made, requires very high skills. Several doses/currents/voltages were tested during the fabrication process, while the parameters of a voltage of 30 kV and a beam current of 1.5 pA was found to be optimal.

The proposed method was experimentally investigated on several ion irradiation doses, but the parameters mentioned above were found to be optimal in order to properly self assemble the nano metric particles. Several sophisticated nano structures, such as the one mentioned in Figure 2, as well as different periods of time between the FIB operation and the self assembly process were tested. The self assembly of the nano metric particles was carried out approximately eight months after generating the charge pattern using the FIB. This long period of time, between the generation of the charged pattern and the self assembly, indicates the high stability as well as the high reliability of creating electronic and photonic devices based on the aforementioned self assembly method.

**Figure 3.**
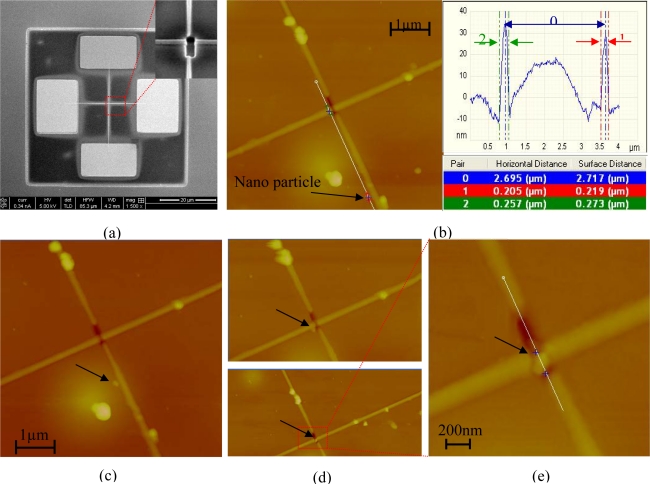
Nano metric electronic transistor. **(a)** Top view SEM image of the fabricated device (including the four electric contacts). **(b)** AFM topographic image of the device. Initial state of the gold nano particle (left panel). A cross Section of the width of the electric transistor, the dimension of the gold nano particle and the horizontal distance between the particle and the air gap (right panel). **(c)** and (**d)** Present the furtherance of the gold nanoparticle towards the air gap (marked in the figures by a black arrow). The lower part of (d) presents the final step where the nanoparticle is located inside the air gap between the two nano wires. **(e)** Enlarged image of the lower part of (d).

Figure 3 presents the preliminary experimental results of a novel nano metric electronic transistor that was fabricated using the FIB. The nano metric particle can be positioned in the proper location following the aforementioned self assembly method or by locating it using manipulation of the AFM tip. In this nanotransistor, we present the optional but the less attractive technique, whereby we manipulate a nano particle using the AFM tip. This technique is mentioned in order to emphasize the simplicity as well as the huge benefit of using the aforementioned self assembly method in multistage devices. Nevertheless, please note also that in our experimental and fabrication attempts we have been able to demonstrate the realization of the proposed self assembly technique (and not by using an AFM) for installation of even a single gold nano particle (e.g., see the lower left corner of the right “Z” structure of Figure 2(a)). To obtain this capability, we had to properly adapt the fabrication process of the structure on the chip as well as to control the concentration of the solution with the nano particles.

In Figure 3(a), we show a top view SEM image of the entire fabricated device, which includes four electric contacts. The two electric vertical contacts control the position of the gold nano metric particle inside an air gap by applying voltage of a few volts across it, whereas the two horizontal contacts act as the input and output ports of the nano metric transistor.

The nano metric transistor was generated by FIB using the FEI Helios NanoLab 600 while applying the following parameters: ion beam voltage of 30 kV and a beam current of 1.5 pA. The fabrication stage of the electrical nano transistor consists of several phases. At first we used the FIB in order to etch part of the silicon layer and to create the square box. At the etching process, we removed about 200 nm from the upper layer of the silicon. Right after the creation of the square box, we deposited the electrodes as well as the nano wires using the FIB with a process called ion beam induced deposition (IBID). The electrodes, as well as the nano wires, are made out of tungsten (W) and they were implanted into the silicon oxide layer. The last phase was creating the required gap between the electrodes. In this stage, we use the FIB in order to etch a small part of the wires and to penetrate into the silicon oxide layer. Therefore, only the gap region is made out of silicon oxide. As mentioned in the article, the self assembly process is achieved only in an insulating material (e.g., the silicon oxide layer), therefore the self assembly process of the nano particles could be self assembled only at the air gap, which is made out of silicon oxide.

In Figure 3(b), we present an AFM topographic image of the device. The left side of Figure 3(b) presents the initial position of the gold nano particle. The right side of Figure 3(b) presents a cross Section along the width of the electronic transistor (denoted in the figure as pair #2) as well as verifies that indeed a 200 nm particle was positioned in an initial position with proper proximity to the air gap (denoted in the figure as pair #1). The horizontal distance between the initial state of the nano particle and the air gap was 2.695 μm and it is denoted in the figure as pair #0.

We used an AFM tip in order to locate the gold nano particle inside the designated air gap. The advancement of the gold nano particle toward the air gap was carried out using the mechanical “Nano manipulation” mode having the following parameters: x-y velocity of 0.1 μm/s, z- velocity of 10 nm/s and z distance that was selected to be −220 nm. The procedure of pushing the nano particle into the air gap is seen in Figure 3(c),(d). Figure 3(e) presents an enlarged image of the lower part of Figure 3(d), where the gold nano particle is already located inside the air gap. The position of the gold nano particle is denoted in the figure by a black arrow.

The AFM measurements and imaging were carried out using the Nanoscope V Multimode scanning probe microscope. All images were obtained using the tapping mode with a single LTSP silicon probe (force constant of 48 N/m, Digital Instruments). The resonance frequency of this cantilever was approximately 167 kHz, while the scan angle was maintained at 0°. The images were captured in the retrace direction with a scan rate of 0.5 Hz. The image resolution is 256 samples /line. After obtaining the measurements, the “planefit” and “flatting” functions were applied to each image of the sample. The dimensions of the particles were determined by an analysis of the phase and height of the extracted AFM images that were collected simultaneously using the Nanoscope Software Version 7.3.

**Figure 4.**
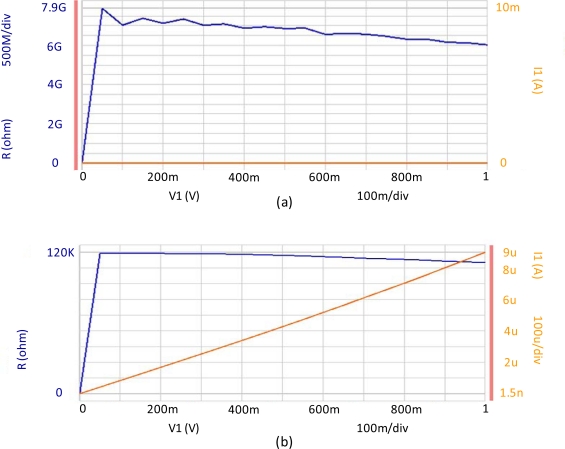
R-V curve of the nano metric device, where the gold nanoparticle is being placed outside **(a)** and inside **(b)** the air gap, respectively (blue lines; left Y- axis). The orange curve presents the I-V (current-voltage) plot (right Y- axis). The horizontal X- axis is the same for both plots.

In order to verify our assumption that indeed the nano particle was inserted into the air gap, and that the proposed nano transistor is functional, we measured the R-V (resistance-voltage) curves for two possible cases. The R-V curves were generated using the Agilent B1500A semiconductor device analyzer. The Y-axis on the left and right side of Figure 4 presents the resistivity and the current flow of the device, respectively, as a function of the voltage that was applied across the vertical pair of electrodes.

In Figure 4(a),(b), we present the R-V curves where the gold nano particle is located outside and inside the air gap, respectively. One may see that when the particle is shifted and positioned inside the air gap between the two nano wires (by the two control electrodes), the current increases *versus* the applied voltage *i.e.*, the electric current is able to flow along the device. When the nano particle is shifted away from the air gap (by applying the proper voltage across the control nano wire electrodes) the current remains zero *i.e.*, no current flow is measured through the device. Note that the nano particles that are located on top of the electrode are relatively large. In essence, each one of them is an aggregate of several nano particles. In the experimental validation of the electronic nano transistor device, these aggregates do not move when external voltage is applied due to their relatively large size. The device can be switched as long as the nano metric particle remains charged. In our internal investigation and based on some other devices that were fabricated based on focused ion beam technology, the nano particle remained charged for about one year. However, we believe from our preliminary experimental validation that the proposed technique can yield devices capable of switching over periods of several years when properly fabricated.

We named the device mentioned in Figure 3 as a nano metric electronic transistor due to its ability to change the current flow between two pairs of terminals (drain and source) when applying a voltage command between the other pair of terminal (gate), as well as due to its ability to switch electronic signal. For instance, in MOSFET or JFET transistors, a voltage needs to be constantly applied between the gate and the source terminals (above the threshold) in order to create the channel, which allows the current to flow from the drain to the source (N-MOS transistor) or in an opposite direction. However, in the proposed nano metric transistor, it may be enough to apply a one single voltage command over the proper terminal (gate) in order to move the nano particle to the proper location inside the air gap and to allow the current to flow between the two pairs of terminals (drain and source). After the nano particle is already located inside the air gap, no additional external voltage is required to maintain the current flow. Therefore, the proposed device is a nano transistor having similar structure to MOSFETs or JFETs, but it also has an important advantage over them is due to its bi-stability property, which can be demonstrated in reduced power consumption.

Moreover, please note that the value of the current that flows between the source and the drain is directly dependent on the overlap existing between the nano metric particle, which is located inside the air gap, and the two pairs of contacts of the source-drain terminals (the value of this current is increased when the overlapping between the nano particle and the two pairs of terminals is increased).

The device can be switched for endless time, as long as the nano metric particle remains charged. In our internal investigation of similar devices, we have been able to fabricate devices where the installed nano particle remained charged for about one year.

Note that in Figure 4, the R-V curve is presented by the blue lines (left Y- axis). The orange curve, on the other hand, presents the I-V (current-voltage) plot (right Y- axis). The horizontal X- axis is the same for both plots.

Due to the self assembly property of the nano particles that was previously demonstrated, one may fabricate plurality of such nano transistor devices in parallel and on top of the same chip. In order to demonstrate the applicability of this method, we generated a nano transistor based on manipulation of 200 nm gold particles. The same technique can be used with much smaller particles (e.g., 5 nm and even smaller) in order to create very small, as well as very attractive (from the fabrication point of view), nano transistors. Therefore, the proposed technique can generate nano transistors going toward dimensions of few nanometers and which are much smaller than those obtained by techniques such as electromigration or electron beam lithography.

In order to experimentally validate the proposed devices, we fabricated more than twelve such devices with different sizes of air gap as well as with different thicknesses of the conductive wires. Therefore the proposed technique may also be reproducible.

**Figure 5.**
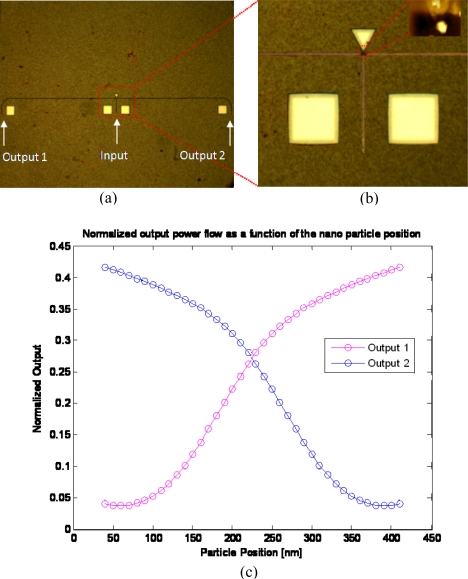
Fabricated “T-junction”: **(a)** Top view microscopic image of the overall fabricated device. **(b)** Enlarged image showing the input waveguide, which splits into a pair of output wave guiding ports. The inset shows an AFM picture of the location of the gold nano particle inside the air gap. **(c)** Simulation result of the normalized outputs as a function of the position of 80 nm particle.

**Figure 6.**
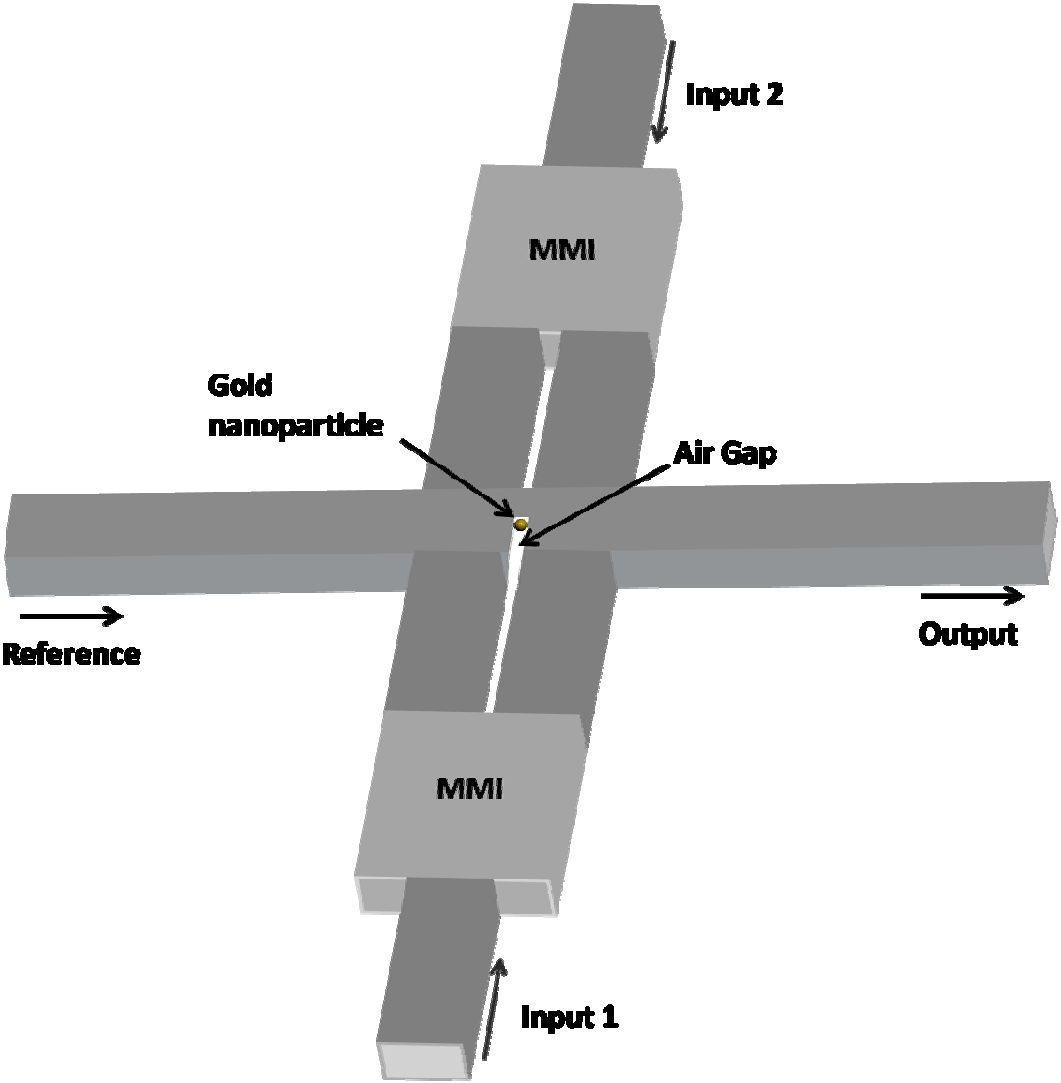
A schematic sketch of the photonic modulator.

Other examples where the discussed self assembly technique becomes useful can be seen in Figures 5 and 6. In those figures, we present other types of tunable nano device that can also benefit from the self assembly property (to allow mass production realization of plurality of such devices on the same chip). Figure 5 shows the top view of a microscope image of a tunable T-junction based photonic switch constructed from silicon (Si) waveguides. The device is aimed to control the direction of wave that propagates along the waveguide. The selectivity of the direction is determined by a gold nano metric particle having diameter of few tens of nanometers. The tunability mechanism works in the following way: an electromagnetic wave at wavelength of 1.55 μm is excited by the laser and propagates along the main waveguide until it approaches the splitting point in the T-junction. A square hole of air of 450 nm is produced by the FIB at the splitting point. The silicon waveguides consist of a wave-guiding channel with a height of 450 nm and width of 250 nm. When the electromagnetic wave hits the nano particle, the energy is reflected, absorbed and scattered. Therefore, shifting the position of the particle inside the air gap may redirect the input beam into either the right or the left output ports (depending on the position of the particle). Figure 5(a) shows the top view microscope images of the fabricated “T-junction” device. Figure 5(b) shows an enlarged image of the relevant splitting point, while the inset picture presents an AFM image of a gold nano particle that is located inside the air gap. In Figure 5(c), we present simulation results of the normalized power flow for the two outputs (the left and the right waveguides of the “T-junction”) *versus* the position of the nano particle inside the air gap. One may see that shifting the nano particle a distance of about 350 nm switches the direction of the signal from the right output (Output 2) to the left output (Output 1) of the “T-junction” device and *vice versa*.

In Figure 6, we present another device that has several functionalities e.g., being an all optical modulator. The conceptual sketch of the device is seen in Figure 6. An air hole with the dimensions 80 nm × 265 nm is fabricated inside the reference waveguide between two multi-mode interference (MMI) regions. On the left side, we have a reference beam that propagates along the waveguide and is coupled to the output of the device. On the upper and lower part, we have the two photonic inputs that control the position of the gold nano particle inside the air gap (by applying optical forces as occurring in optical tweezers [21]).

In this case, the main advantage of the aforementioned self assembly method, is to have the ability to cascade a huge number of such photonic devices and to locate each nano metric particle in the proper predetermined position.

## 4. Conclusions

In this paper, we have presented the use of focused ion beam for generating well-defined arrangement of self assembly nano particles into plurality of sophisticated patterns. The self assembly method relies on implantation of positive gallium ions that acts as an intermediary layer between the nano particles and the surface. After positioning the nano particles, the proposed devices can be used as electronic nano transistors, electro-optical nano switches or nano photonic all optical modulators.

The fabrication procedure for the novel nano metric devices, the self assembly of the nano particles, as well as the experimental characterization of the nano transistor, were demonstrated and discussed.
